# Polar Coordinate Analysis of Relationships With Teammates, Areas of the Pitch, and Dynamic Play in Soccer: A Study of Xabi Alonso

**DOI:** 10.3389/fpsyg.2018.00389

**Published:** 2018-03-23

**Authors:** Rubén Maneiro Dios, Mario Amatria Jiménez

**Affiliations:** Faculty of Science of Education, Pontifical University of Salamanca, Salamanca, Spain

**Keywords:** performance analysis, observational methodology, soccer, polar coordinates, Xabi Alonso

## Abstract

Research in soccer has traditionally focused on very specific aspects of the game, such as technical and physiological aspects, and has largely ignored important issues such as tactical performance and the role of individual players within the team. The aim of this study was to study the different relationships that Xabi Alonso, one of the world's best midfielders, establishes with his teammates during offensive play, and to investigate his connections with the pitch in terms of where his direct interventions started and finished, his use of technical actions, his involvement in set plays and interceptions, and his relationship with shots at goal. To do this, we analyzed all the matches played by the winner of the 2012 UEFA European Championship: Spain. We employed an observational methodology design (Anguera, 1979) using a modified version of the *ad hoc* soccer observation instrument designed by Amatria et al. (2016). The resulting data were analyzed by polar coordinate analysis (Gorospe and Anguera, 2000), which is a powerful data reduction technique with high predictive power. The results showed significant associations (*Z* > 1.96; *p* < 0.05) between Alonso and players in different positions, a wide sphere of influence on the pitch, both for the start and end of interventions, and a strong link with game interruptions and interceptions and with the use of different technical actions. No significant associations were detected for type of shot. Studies on tactical performance that take account of the multiple factors involved in soccer will lead to better decision-making by coaches and facilitate analysis of a player's true performance.

## Introduction

Research in soccer has traditionally focused on more mechanical aspects of the game, such as training, biomechanics, health and injury, and physiology. Tactical studies came later, with early studies typically analyzing isolated quantitative data that had little to offer to a game such as soccer, which is marked by constant change and a high degree of uncertainty (Svensson and Drust, 2005). The emergence of increasingly sophisticated data analysis software, however, combined with the development of rigorous methodological approaches, gave researchers the tools to prove or disprove numerous theories about soccer that had been hitherto lacking in scientific credibility.

One particularly prominent area of current research in the field of soccer is how players interact with each other on the pitch (Carling et al., 2008; Duch et al., 2010; Ric et al., 2017). The depth and sophistication of these interactions, which are directly linked to creativity and intra-team coordination (Furley and Memmert, 2015), confirm that soccer, together with its underlying structures, is a complex system (McGarry et al., 2002).

On the whole, complex data are easier to analyze when broken down into measurable units (Bakeman and y Quera, 1996). In the case of soccer, this “simplification” is the only way to validate associations and/or causal relationships that occur during play and to gain insights into behaviors and interactions from a certain distance. In this complex game, the whole is greater than the sum of its parts, hence the importance of understanding how different players interact and relate to each other. One of the main data analysis techniques for analyzing complex interactions in soccer is polar coordinate analysis applied to data gathered through systematic observation (Anguera and Hernández-Mendo, 2015; Castañer et al., 2016, 2017). Polar coordinate analysis can be used to measure the spontaneous behavior of players interacting in their natural environment from the perspective of a given behavior, known as a *focal behavior*.

Polar coordinate analysis has proven to be an effective tool for breaking down the complexity of the game (Lago and Anguera, 2002; Castellano and Hernández-Mendo, 2003; Perea et al., 2012; Robles et al., 2013), and its application to the study of two of the world's best strikers, Lionel Messi (Castañer et al., 2016) and Cristiano Ronaldo (Castañer et al., 2017), has enabled researchers to draw practical conclusions on what makes these players great and in turn to make recommendations for improving both offensive and defensive aspects of play.

Polar coordinate analysis, however, has not yet been tested in midfield players. The bulk of research on midfielders draws on subjective opinions based on both quantitative (Taylor et al., 2005) and qualitative (Wiemeyer, 2003; Thelwell et al., 2006) data analyzed out of context, i.e., with no consideration of other inputs, such as interactions with the ball or other players or the strategic use of space.

Relationships between players are typically visualized through a lens focused on their traditional role according to their position on the pitch. Midfielders are “permeable” to the flow of information from other players on their team; they are a central driving force, positioned at the crossroads between attack and defense. In a recent study on leadership roles in sport, Fransen et al. (2016) showed that players with central positions had a privileged position, which combined with their high tactical responsibilities, positioned them as team leaders. Soccer coach Jürgen Klopp, referring to a Champions League match in 2014, said the following about rival midfielder Xabi Alonso: “Our plan was to take Xabi out of the game. Because if Alonso can play as he wants it is impossible to defend against Madrid.”

There have been recent claims that midfielders are the most important members of a soccer team (Duch et al., 2010), particularly at the start and end of an attack (Clemente et al., 2015), due to the highly specific and versatile nature of their position (Kannekens et al., 2010). Additional requirements of midfielders include the combination of sophisticated motor (Di Salvo et al., 2009; Carling et al., 2010) and technical skills (Bloomfield et al., 2007), even in situations when time is critical (Rampinini et al., 2007).

Considering the above, we believe that an in-depth analysis of one of the world's greatest midfield players, Xabi Alonso, in his natural environment through the study of interconnections between multiple, suitably coded, behaviors, is justified. The application of a robust methodological approach, combined with an in-depth, multidimensional analysis of rigorously coded data, will help to provide objective insights into how Alonso interacts with his environment and makes him unique. The specific aim was to analyze Alonso's spatial, technical, and tactical skills during the course of play together with his interactions with the other players on his team.

## Method

### Design

We undertook an systematic observation study (Anguera, 1979). Observational methodology is currently considered to be one of the most suitable methodologies for analyzing spontaneous interactions in sport (Castellano et al., 2012; Anguera and Hernández-Mendo, 2015).

The specific design employed was I/P/M, which stands for Idiographic, Point, and Multidimensional. It was *idiographic* (Anguera et al., 2011) because we studied a single player, *point*, because we applied intrasessional follow-up only (Sánchez-Algarra and Anguera, 2013), and *multidimensional*, because we analyzed behaviors from different dimensions in the observation unit. The observation of behavior was scientifically rigorous because the events observed were fully perceivable and the observers had a non-participatory role.

### Participants

The observation sample was a convenience sample (Anguera et al., 2011) formed by a single player, Alonso, during his participation in the final phases of the 2012 UEFA European Championship (UEFA Euro 2012) as a member of the national Spanish team.

As such, the study can be considered a case study (Yin, 2014; Castañer et al., 2016). The behaviors were annotated by analyzing video footage of the matches broadcast on public television. The study thus complies with the ethical principles of the Declaration of Helsinki.

### Observation instrument

**Figure 1:**
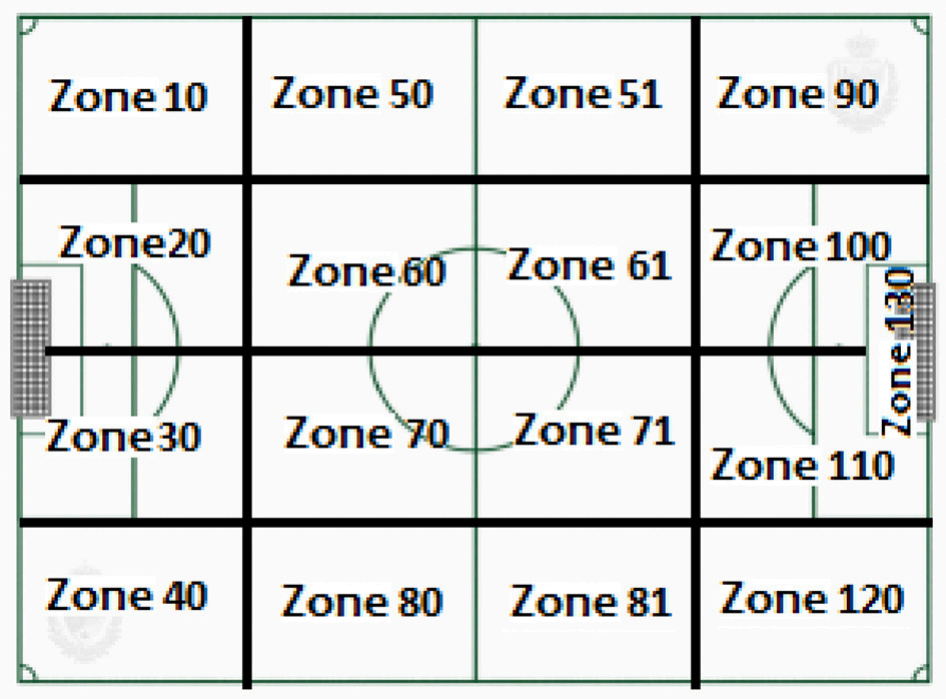
Pitch areas.

**Figure 2:**
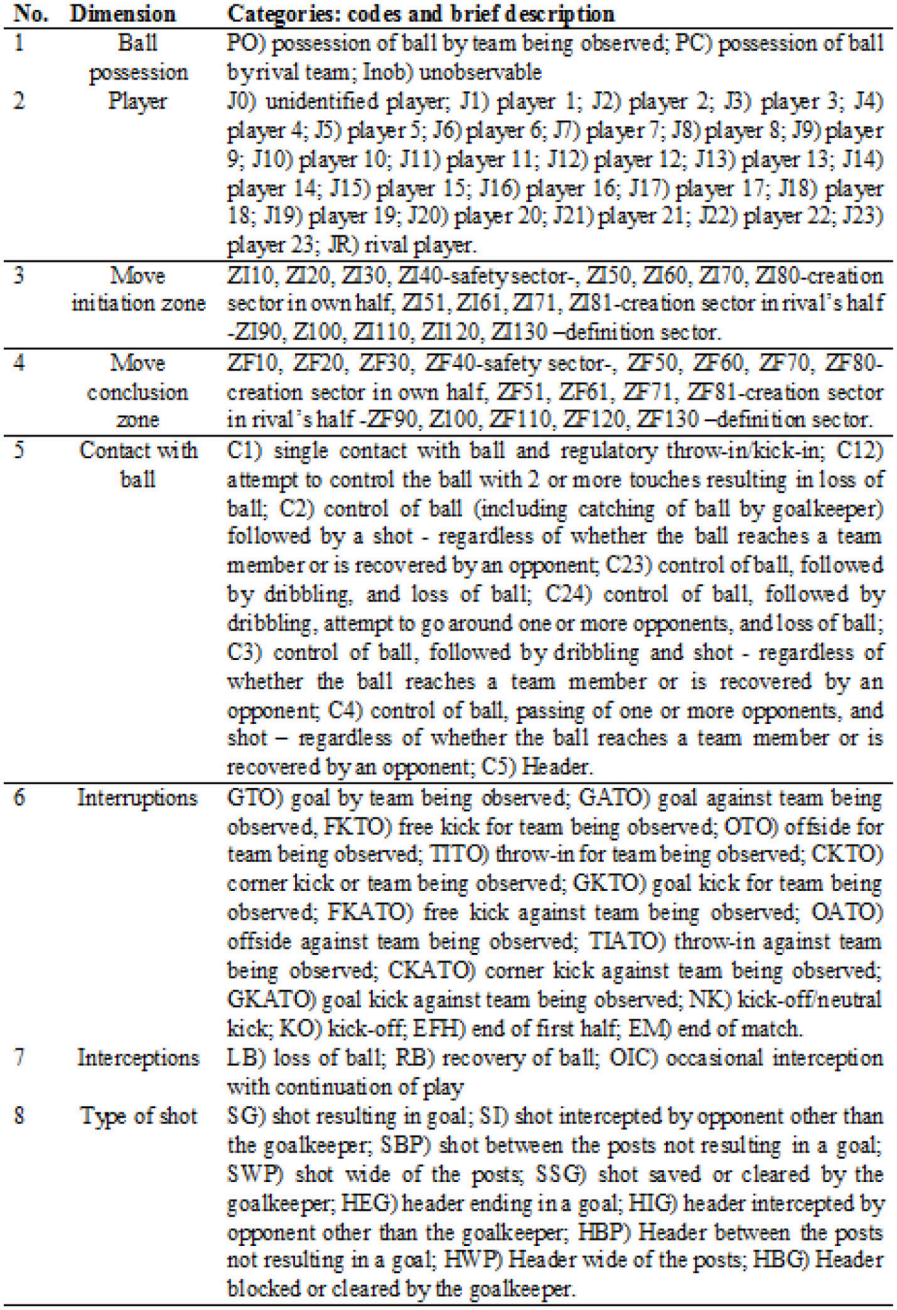
Observation instrument. Source: Modified from Amatria et al. (2016).

We created a modified version of the original *ad hoc* soccer observation instrument built by Amatria et al. (2016). The modifications included new subdivisions that reflected the length of the pitch (Figure 1) and more specific definitions of players, technical actions, types of shot, and interruptions (e.g., set plays; Figure 2).

The instrument is a combination of a field format and systems of categories (Anguera et al., 2007). It contains eight dimensions, each of which is broken down into a system of exhaustive, mutually exclusive categories.

### Data annotation and coding

The data from the video footage were annotated (Hernández-Mendo et al., 2014) using the freely available software program LINCE (v. 1.2.1; Gabín et al., 2012). The interobserver agreement analysis yielded a kappa value of 0.95. The data were concurrent, time-based (type IV) data (Bakeman, 1978).

Two additional software programs were used: GSEQ v5.1 (Bakeman and Quera, 2011) for the lag sequential analysis and HOISAN v. 1.2 (Hernández-Mendo et al., 2012) for the polar coordinate analysis. Lag sequential analysis is needed to calculate the adjusted residual values necessary for polar coordinate analysis.

### Data analysis

Polar coordinate analysis was developed by Sackett (1980) and later improved by Anguera (1997). Although conceptual (Anguera and Losada, 1999) and empirical (Hernández-Mendo and Anguera, 1998, 1999; Gorospe, 1999; Gorospe and Anguera, 2000; Castellano and Hernández-Mendo, 2002) studies started using this method decades ago, its use in sports sciences is recent (Anguera and Hernández-Mendo, 2015). Polar coordinate analysis has attracted increasing attention in recent years, as it offers numerous features that are ideal for the type study proposed in this paper (González et al., 2013; Robles et al., 2014; Echeazarra et al., 2015; López-López et al., 2015; Morillo-Baro et al., 2015; Sousa et al., 2015; Castañer et al., 2016, 2017; López et al., 2016; Aragón et al., 2017; Tarragó et al., 2017; Prudente et al., 2018).

The starting point of any polar coordinate analysis study is the calculation of adjusted residuals using lag sequential analysis (Bakeman, 1978). These are calculated both prospectively (for each positive lag considered) and retrospectively (for each negative lag considered). Following the proposal of Sackett (1980), these residuals are then standardized and used to calculate Zsum statistics (Cochran, 1954), which, in turn, are used to produce a vector map showing the statistical relationships between the behavior of interest, known as the *focal behavior*, and other behaviors, known as *conditional behaviors*.

The *Z*_*sum*_ statistic is calculated using the formula $Z_{sum}=\frac{\sum Z}{\sqrt{n}}$, where *Z* corresponds to each of the standardized adjusted residuals reflecting the relationship between the focal behavior and a conditional behavior at each of the lags considered, and where n is the number of lags. The *Z*_*sum*_ statistic is calculated separately for each prospective and retrospective lag, thereby resulting in a prospective and retrospective value for each conditional behavior. The relationship between the focal behavior and each of the conditional behaviors is depicted by the length and angle of the corresponding vectors (Sackett, 1980).

The length of each vector is equivalent to the hypotenuse of a right-angled triangle in which the respective prospective and retrospective *Z*_*sum*_ values correspond to the lengths of the sides adjacent to the right angle, in other words, $Length=\sqrt{{\left(ZsumP\right)}^{2}+{\left(ZsumR\right)}^{2}}$. The angle is calculated via a trigonometric function, by which $\phi =\frac{arc\;sen\;ZsumR}{Length}$.

The resulting value (ϕ) is then transformed according to the quadrant in which the vector is located. The position of the vector is determined by the interplay between the positive and/or negative signs carried by the prospective and retrospective *Zsum* values, which are respectively plotted along the X and Y axes.

**Figure 3:**
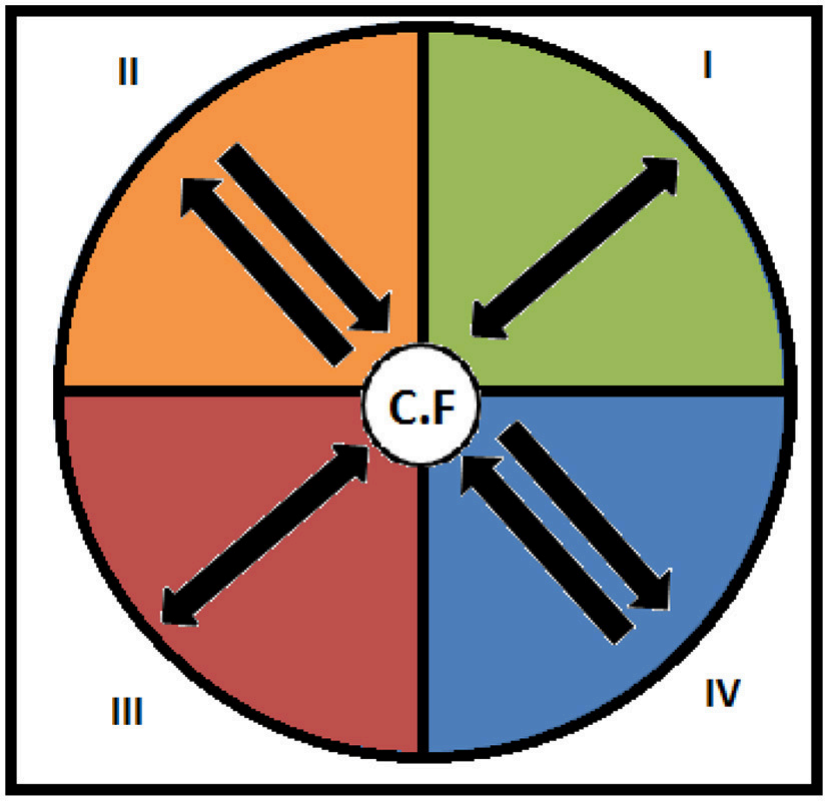
Activating and inhibitory relationships between focal and conditional behaviors.

The above calculations are performed in HOISAN (López-López et al., 2015), which also produces the results in the form of easy-to-interpret vector maps. Vectors located in quadrant I show focal behaviors that activate and at the same time are activated by conditional behaviors; those located in quadrant II show focal behaviors that inhibit but are not inhibited by conditional behaviors; those located in quadrant III show focal behaviors that inhibit and at the same time are inhibited by conditional behaviors; and those located in quadrant IV show focal behaviors that activate but are not activated by conditional behaviors (Figure 3).

## Results

As mentioned in the Methods section, before performing the polar coordinate analysis, we first applied lag sequential analysis to investigate the statistical associations between the categories from the observation instrument.

We have grouped our results into six sections, each describing a different aspect of Alonso's performance and interaction with his teammates and environment during UEFA Euro 2012:

- Relationship between Alonso and other team members
- Relationship between Alonso and intervention initiation zone
- Relationship between Alonso and intervention conclusion zone
- Relationship between Alonso and game situations (interruptions and interceptions)
- Relationship between Alonso and technical action (ball contact)
- Relationship between Alonso and type of shot he takes

Alonso (code J14) was established as the focal behavior for all the above analyses.

### Relationship between alonso and other players

For this analysis, we investigated the relationship between Alonso/J14, defined as the focal behavior or category, and the other players on the Spanish national team—J0 (unidentified player), J1 (Iker Casillas), J2 (Raúl Albiol), J3 (Gerard Piqué), J4 (Javi Martinez), J5 (Juanfran), J6 (Iniesta), J7 (Pedro), J8 (Xavi Hernández), J9 (Fernando Torres), J10 (Cesc Fábregas), J11 (Álvaro Negredo), J12 (Víctor Valdés), J13 (Juan Mata), J15 (Sergio Ramos), J16 (Busquets), J17 (Arbeloa), J18 (Jordi Alba), J19 (Fernando Llorente), J20 (Cazorla), J21 (David Silva), J22 (Jesús Navas), and J23 (Reina)—and rival players (JR), defined as the conditional behaviors or categories. The aim was to investigate how Alonso interacted with teammates and rivals during the matches analyzed in UEFA Euro 2012.

**Figure 4:**
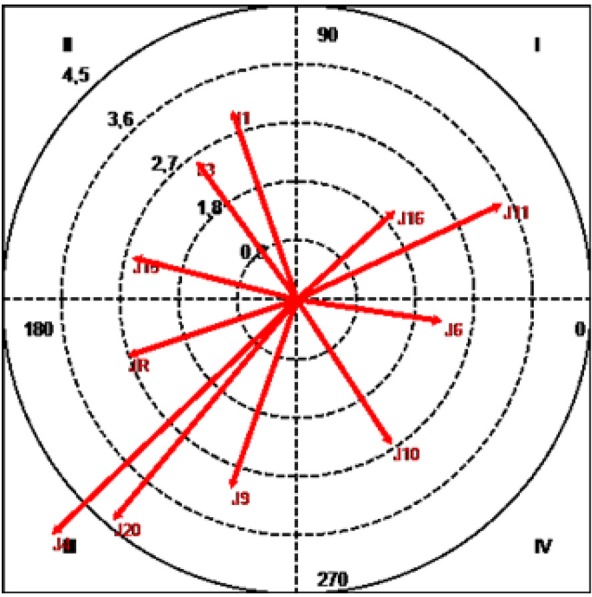
Vector map showing relationships between focal category (Alonso/J14) and other players.

The results (Table 1 and Figure 4) show that both J11 (Álvaro Negredo), with a radius of 3.44 and an angle of 25.07°, and J16 (Sergio Busquets), with a radius of 2.02 and an angle of 42.13, were located in quadrant I, where the focal behavior activates the conditional behavior both prospectively and retrospectively (mutual activation).

**Table 1:** Polar coordinate analysis results for the relationship between the focal category Alonso (J14) and other players.

**Category**	**Quadrant**	**Prospective perspective**	**Retrospective perspective**	**Radius**	**Angle**
J0	IV	0.34	−0.11	0.36	342.01
J1	II	−0.99	2.89	3.05^*^	108.89
J3	II	−1.53	2.11	2.61^*^	126.06
J4	III	−3.73	−3.58	5.17^*^	223.84
J6	IV	2.22	−0.33	2.25^*^	351.53
J7	IV	0.97	−0.6	1.14	328.42
J8	I	0.95	0.11	0.96	6.69
J9	III	−1.01	−2.88	3.05^*^	250.63
J10	IV	1.44	−2.21	2.64^*^	302.96
J11	I	3.12	1.46	3.44^*^	25.07
J13	II	−0.72	1.77	1.91	112.18
J15	II	−2.51	0.64	2.59^*^	165.7
J16	I	1.5	1.36	2.02^*^	42.13
J17	IV	0.5	−0.05	0.5	353.83
J18	I	1	1.37	1.69	53.92
J20	III	−2.8	−3.36	4.38^*^	230.14
J21	IV	0.73	−0.72	1.03	315.53
J22	I	0.13	0	0.13	0
JR	III	−2.58	−0.86	2.72^*^	198.41

* *p < 0.05*.

J1 (Iker Casillas), with a radius of 3.05 and an angle of 108.89°, J3 (Gerard Piqué), with a radius of 2.61 and an angle of 126.06°, and J15 (Sergio Ramos), with a radius of 2.59 and an angle of 165.7°, were positioned in quadrant II, where the focal behavior inhibits but is not inhibited by the conditional behavior.

Quadrant III contains the conditional categories J4 (Javi Martinez), J9 (Fernando Torres), J20 (Cazorla), and JR (rival player), with respective radii of 5.17, 3.05, 4.38, and 2.72, and respective angles of 223.84, 250.63, 230.14, and 198.41°. In this quadrant the focal and conditional behaviors are mutually inhibited.

Finally, J6 (Iniesta), with a radius of 2.25 and an angle of 351.53°, and J10 (Cesc Fábregas), with a radius of 2.64 and an angle of 302.96°, were located in quadrant IV, where the focal behavior activates but is not activated by conditional behaviors -ZI71 and ZI10-.

### Relationship between alonso and intervention initiation zone

In this analysis, we investigated the relationship between Alonso (J14) and the different areas of the pitch in which an intervention involving this player was launched (ZI10, ZI20, ZI30, ZI40, ZI50, ZI60, ZI70, ZI80, ZI51, ZI61, ZI71, ZI81, ZI90, ZI100, ZI110, ZI120, and ZI130). The aim was to identify Alonso's use of space during the course of play.

**Figure 5:**
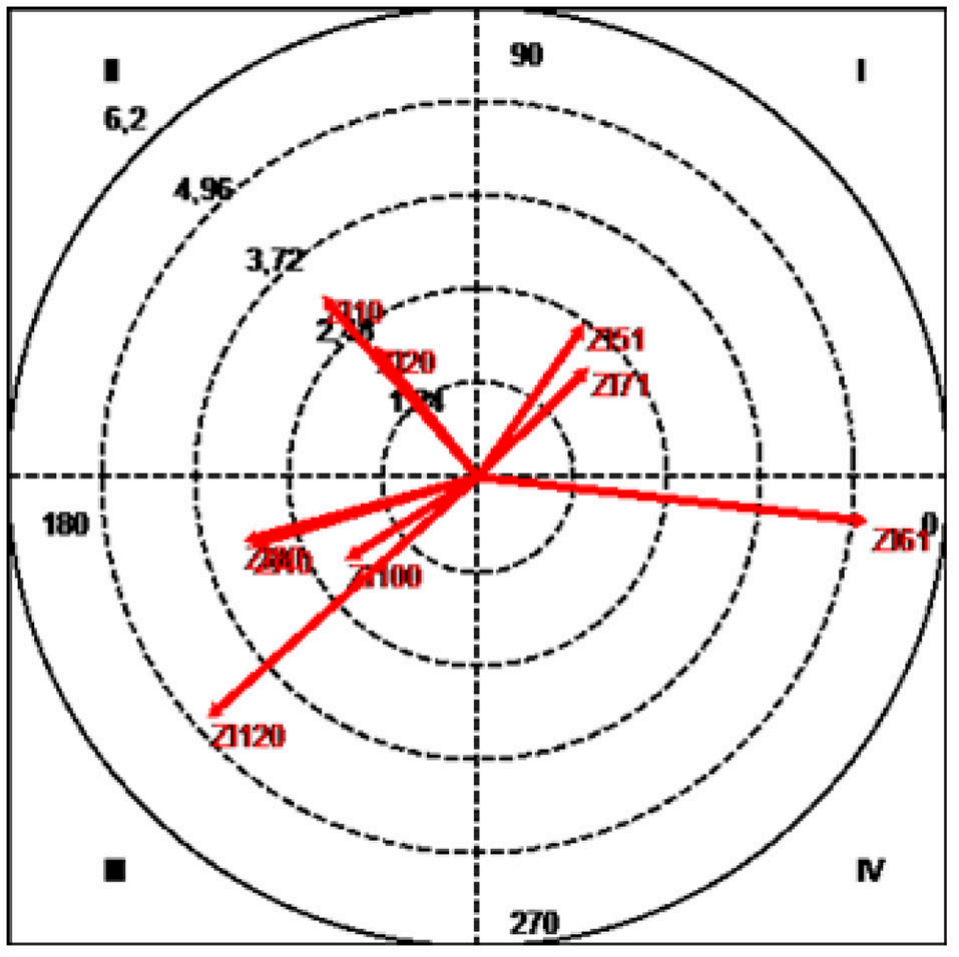
Vector map showing relationships between the focal category (Alonso/J14) and intervention initiation zones.

The results (Table 2 and Figure 5) show that both ZI51, with a radius of 2.49 and an angle of 55.17°, and ZI71, with a radius of 2.07 and an angle of 44.74°, were located in quadrant I (mutual activation between focal and conditional behaviors).

**Table 2:** Polar coordinate analysis results for the relationship between the focal category Alonso (J14) and intervention initiation zones.

**Category**	**Quadrant**	**Prospective perspective**	**Retrospective perspective**	**Radius**	**Angle**
ZI10	II	−2.06	2.41	3.18^*^	130.49
ZI20	II	−1.37	1.74	2.21^*^	128.35
ZI30	II	−1.83	0.04	1.83	178.6
ZI40	III	−2.99	−0.9	3.12^*^	196.75
ZI50	I	0.34	0.46	0.57	53.67
ZI60	IV	0.28	−0.65	0.71	293.34
ZI70	I	1.45	0.21	1.47	8.23
ZI80	III	−3.11	−0.85	3.22^*^	195.29
ZI51	I	1.42	2.04	2.49^*^	55.17
ZI61	IV	5.18	−0.59	5.21^*^	353.5
ZI71	I	1.47	1.46	2.07^*^	44.74
ZI81	IV	0.82	−0.37	0.9	335.72
ZI90	III	−1.5	−1.26	1.96^*^	220.09
ZI100	III	−1.76	−1.1	2.07^*^	211.94
ZI110	IV	0.08	−0.8	0.81	275.71
ZI120	III	−3.58	−3.2	4.8^*^	221.83
ZI130	II	−0.64	1.04	1.22	121.72

* *p < 0.05*.

Quadrant II, which shows conditional behaviors that are inhibited by but do not inhibit the focal behavior, contains the two zones located to the left of the Spanish team's goal: ZI10, with a radius of 3.18 and an angle of 130.49°, and ZI20, with a radius of 2.21 and an angle of 128.35°.

Quadrant III (mutual inhibition quadrant) contained categories ZI40, with a radius of 3.12 and an angle of 196.5°; ZI80, with a radius of 3.22 and an angle of 195.29°; ZI90, with a radius of 1.96 and an angle of 220.09°; ZI100, with a radius of 2.07 and an angle of 211.94°; and ZI120, with a radius of 4.8 and an angle of 221.83°.

Finally, ZI61, with a radius of 5.21 and an angle of 353.5°, was located in quadrant IV, where the focal category activates but is not activated by the conditional behavior.

### Relationship between alonso and initiation conclusion zones

In this analysis, we studied the relationship between Alonso (J14) and the different areas of the pitch in which interventions in which he was involved ended (ZF10, ZF20, ZF30, ZF40, ZF50, ZF60, ZF70, ZF80, ZF51, ZF61, ZF71, ZF81, ZF90, ZF100, ZF110, ZF120, and ZF130). The aim was to analyze how Alonso interacted with these zones when the Spanish team was attacking.

**Figure 6:**
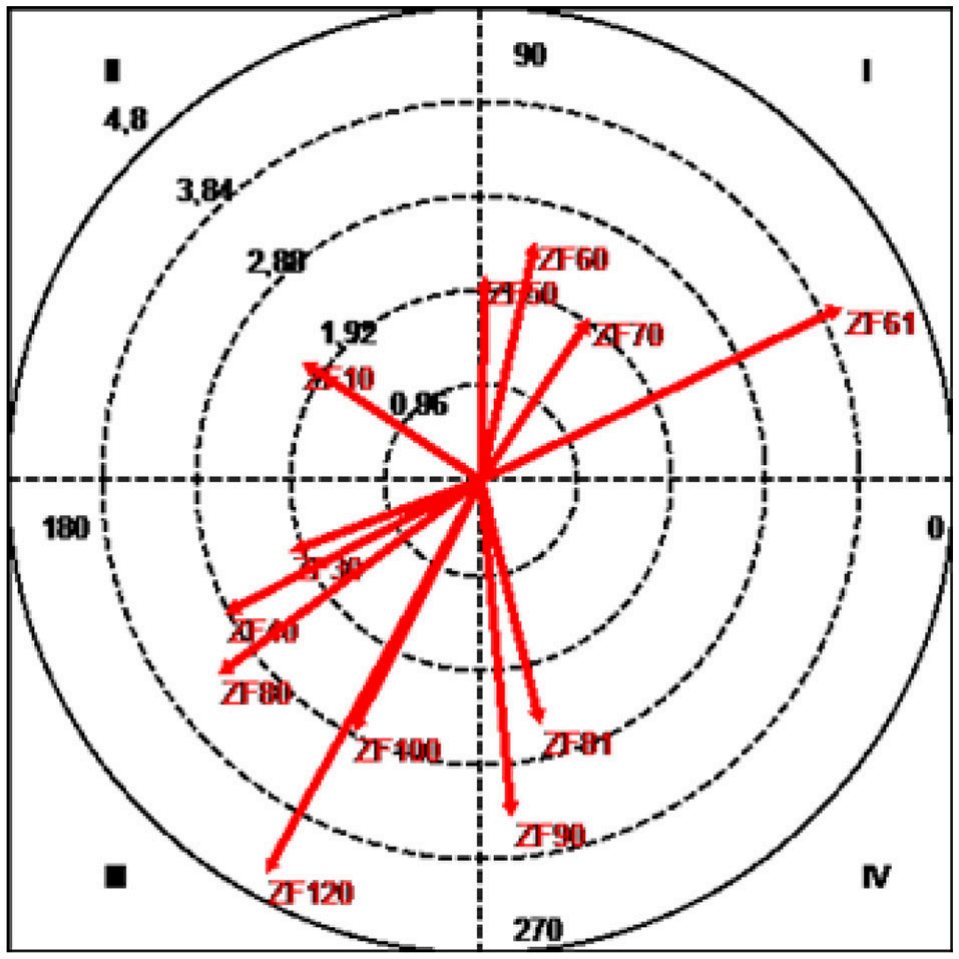
Vector map showing relationships between focal category (Alonso/J14) and intervention conclusion zones.

The results (Table 3 and Figure 6) show that quadrant I (mutual activation) contained categories ZF61, with a radius of 4.05 and an angle of 25.63°; ZF50, with a radius of 2.07 and an angle of 89.01°; ZF60, with a radius of 2.47 and an angle of 77.22°; and ZF70, with a radius of 1.98 and an angle of 56.17°.

**Table 3:** Polar coordinate analysis results for the relationship between the focal category Alonso (J14) and intervention conclusion zones.

**Category**	**Quadrant**	**Prospective perspective**	**Retrospective perspective**	**Radius**	**Angle**
ZF10	II	−1.81	1.2	2.17^*^	146.51
ZF20	II	−1.38	0.52	1.48	159.26
ZF30	III	−1.93	−0.72	2.06^*^	200.56
ZF40	III	−2.58	−1.34	2.91^*^	207.35
ZF50	I	0.04	2.07	2.07^*^	89.01
ZF60	I	0.55	2.41	2.47^*^	77.22
ZF70	I	1.1	1.64	1.98^*^	56.17
ZF80	III	−2.65	−1.96	3.29^*^	216.5
ZF51	I	1.03	1.57	1.88	56.76
ZF61	I	3.65	1.75	4.05^*^	25.63
ZF71	I	0.88	1.74	1.95	63.14
ZF81	IV	0.6	−2.47	2.54^*^	283.62
ZF90	IV	0.31	−3.4	3.42^*^	275.18
ZF100	III	−1.29	−2.54	2.85^*^	243.11
ZF110	IV	0.35	−1.75	1.78	281.28
ZF120	III	−2.17	−3.97	4.53^*^	241.31
ZF130	II	−0.96	0.7	1.19	144.04

* *p < 0.05*.

Quadrant II, in turn, which shows conditional behaviors that are inhibited by but do not inhibit the focal behavior, contained ZF10 (safety sector in team's own half), with a radius of 2.17 and an angle 146.51°.

Quadrant III (mutual inhibition quadrant) contained ZF30, with a radius of 2.06 and an angle of 200.56°; ZF40, with a radius of 2.91 and an angle of 207.35°; ZF80, with a radius of 3.29 and an angle of 216.5°; ZF100, with a radius of 2.85 and an angle of 243.11°; and ZF120, with a radius of 4.53 and an angle of 241.31°.

Finally, quadrant IV, which shows conditional behaviors that are activated by but do not activate the focal behavior, contained ZF81, with a radius of 2.54 and an angle of 283.62, and ZF90, with a radius of 3.42 and an angle of 275.18°.

### Relationship between alonso and game interruptions and interceptions

For this analysis, we analyzed the relationship between Alonso (J14) and different aspects related to game interruptions and interceptions—GTO (goal by team being observed), GATO (goal against team being observed), FKTO (free kick for team being observed), OTO (offside for team being observed), TITO (throw-in for team being observed), CKTO (corner kick or team being observed), GKTO (goal kick for team being observed), FKATO (free kick against team being observed), OATO (offside against team being observed), TIATO (throw-in against team being observed), CKATO (corner kick against team being observed), GKATO (goal kick against team being observed), NK (kick-off/neutral kick), KO (kick-off), EFH (end of first half), EM (end of match), LB (loss of ball), RB (recovery of ball), and OIC (occasional interception with continuation of play)—The aim was to investigate Alonso's involvement in these situations.

**Figure 7:**
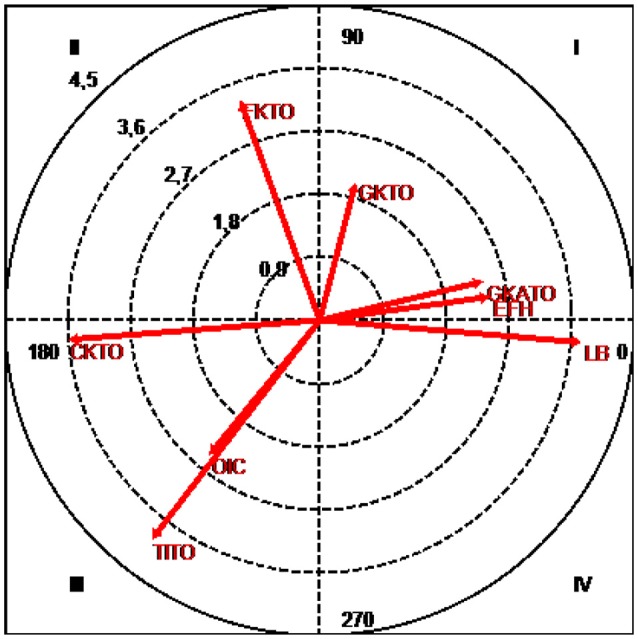
Vector map showing the relationships between the focal category (Alonso/J14) and game situations (interruptions and interceptions).

The results (Table 4 and Figure 7) show that GKTO (goal kick for team being observed), with a radius of 2.07 and an angle of 75.48°; GKATO (goal kick against team being observed), with a radius of 2.44 and an angle of 13.37°; and EFH (end of first half), with a radius of 2.5 and an angle of 7.93°, were all located in the mutual activation quadrant I.

**Table 4:** Polar coordinate analysis results for the relationship between focal category Alonso (J14) and game situations (interruptions and interceptions).

**Category**	**Quadrant**	**Prospective perspective**	**Retrospective perspective**	**Radius**	**Angle**
GTO	II	−0.36	0.51	0.62	125.3
FKTO	II	−1.15	3.18	3.38^*^	109.87
OTO	I	0.5	0.54	0.74	47.21
TITO	III	−2.43	−3.17	3.99^*^	232.55
CKTO	III	−3.64	−0.28	3.65^*^	184.43
GKTO	I	0.52	2	2.07^*^	75.48
FKATO	II	−0.14	0.64	0.66	102.61
OATO	II	−1.83	0.13	1.83	175.8
TIATO	II	−1.2	1.37	1.82	131.12
GKATO	I	2.37	0.56	2.44^*^	13.37
NK	III	−0.19	−1.07	1.09	259.84
NC	I	1.22	0.5	1.32	22.13
EFH	I	2.47	0.34	2.5^*^	7.93
EM	II	−0.52	0.62	0.81	130.29
LB	IV	3.79	−0.32	3.81^*^	355.21
RB	I	1.12	0.47	1.22	22.51
OIC	III	−1.6	−1.95	2.52^*^	230.62

* *p < 0.05*.

Quadrant II, in which conditional behaviors are inhibited by but do not inhibit the focal behavior, contained FKTO (free kick for team being observed), with a radius of 3.38 and an angle of 109.87°.

Quadrant III, where the focal and conditional behaviors mutually inhibit each other, contained TITO (throw-in for team being observed) with a radius of 3.99 and an angle of 232.55°; CKTO (corner kick for team being observed) with a radius of 3.65 and an angle of 184.43°; and OIC (occasional interception with continuation of play) with a radius of 2.52 and an angle of 230.62°.

Finally, the conditional category LB (loss of ball), with a radius of 3.81 and an angle of 355.21°, was located in quadrant IV, where focal behaviors activate but are not activated by conditional behaviors.

### Relationship between alonso and technical actions (ball contact)

For this analysis, we studied the relationship between Alonso and the different categories in the ball contact dimension C1 (single contact with ball and regulatory throw-in/kick-in), C12 (attempt to control the ball with 2 or more touches resulting in loss of ball), C2 (control of ball, including catching of ball by goalkeeper, followed by a shot—regardless of whether the ball reaches a team member or is recovered by an opponent), C23 (control of ball, followed by dribbling, and loss of ball), C24 (control of ball, followed by dribbling, attempt to go around one or more opponents, and loss of ball), C3 (control of ball, followed by dribbling and shot—regardless of whether the ball reaches a team member or is recovered by an opponent), C4 (control of ball, passing of one or more opponents, and shot—regardless of whether the ball reaches a team member or is recovered by an opponent), C5 (header). The aim was to investigate Alonso's technical skills.

**Figure 8:**
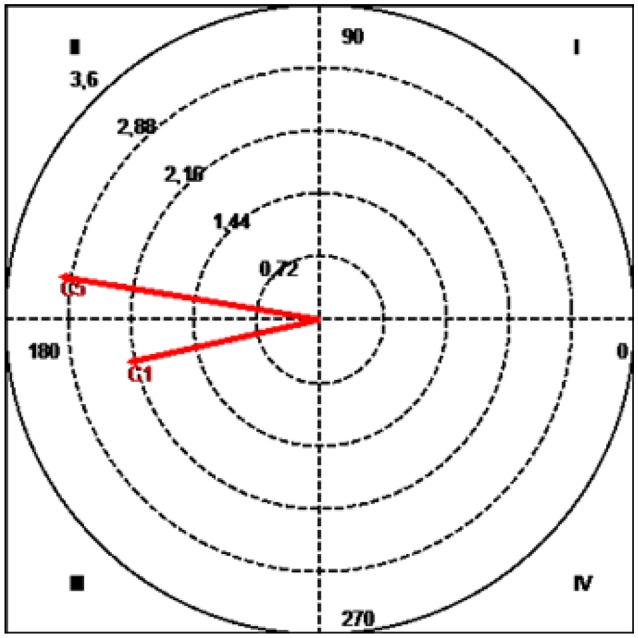
Vector map showing relationships between focal category (Alonso/J14) and type of contact.

Quadrant II, where the focal behavior inhibits the presence of the conditional behavior prospectively and activates it retrospectively, contained the category C5 (header), with a radius of 3.06 and an angle of 170.58° (Table 5 and Figure 8).

**Table 5:** Polar coordinate analysis results for the relationship between the focal category (Alonso/J14) and technical actions.

**Category**	**Quadrant**	**Prospective perspective**	**Retrospective perspective**	**Radius**	**Angle**
C1	III	−2.23	−0.5	2.29^*^	192.65
C12	I	1.36	1.07	1.74	38.2
C2	I	1.04	0.01	1.04	0.49
C23	IV	1.78	−0.26	1.79	351.69
C24	IV	0.11	−0.55	0.57	281.4
C3	IV	1.57	−0.29	1.59	349.64
C4	II	−0.09	1.04	1.04	95.17
C5	II	−3.02	0.5	3.06^*^	170.58

* *p < 0.05*.

The mutual inhibition quadrant, quadrant III, contained the conditional behavior C1 (single contact with ball and regulatory throw-in), with a radius of 2.29 and an angle of 192.65°.

### Relationship between alonso and type of shot

For this analysis, we studied the relationship between Alonso and the different categories in the type of shot dimension (SG, SI, SBP, SWP, SSG, HEG, HIG, HBP, HWP, and HBG). The aim was to identify significant associations between Alonso and the type of shots he takes.

As shown in Table 6, the results were not significant.

**Table 6:** Polar coordinate analysis results for focal category (Alonso/J14) and type of shot.

**Category**	**Quadrant**	**Prospective perspective**	**Retrospective perspective**	**Radius**	**Angle**
SG	III	−1.17	−0.35	1.22	196.58
SI	III	−0.41	−0.99	1.07	247.49
SWP	I	1.21	0.91	1.51	36.84
PT	I	1.16	0.22	1.18	10.71
HEG	III	−0.78	−0.01	0.78	180.65
HIG	II	−0.98	0.04	0.98	177.92
HWP	III	−1.19	−0.1	1.19	184.94
HBG	I	0.46	0.33	0.57	35.7

* *p < 0.05*.

## Discussion

Polar coordinate analysis has been shown on multiple occasions to be an ideal technique for dissecting the complexity underlying the relationships between different players of a team. It is a powerful data reduction technique that produces a manageable set of vectorial parameters that are graphically represented in a vector map that shows both activating and inhibitory relationships between variables of interest (Gorospe and Anguera, 2000). Specifically, the map shows how a given category, the *focal behavior*, is connected to all other categories within a category system. In this vector map, the angle of the vector indicates the nature of the relationship between two behaviors or categories and the radius indicates the strength of the relationship (Anguera and Hernández-Mendo, 2015; Morillo-Baro et al., 2015).

Most studies of performance in soccer to date have focused on the performance of the team as a whole, with little attention given to individual performance or interactions between team members. The most notable studies to date of individual performance are two studies by Castañer et al. (2016, 2017) that used polar coordinate analysis to study and compare Messi and Ronaldo's use of motor skills in relation to goal scoring. Methodologically rigorous studies of the tactics used by midfielders are also lacking. As pointed out by Sampaio and Maças (2012), Memmert (2010) ,Memmert et al. (2017) and Castañer et al. (2016, 2017), in order to understand the performance of a team as a whole, it is first necessary to understand how the different members of the team interact with each other.

### Alonso and his team mates

Our analysis revealed mutual activation between Alonso (J14) and both Negredo (J11) and Busquets (J16), showing that Alonso's interactions with his teammates extend beyond the midfield area into both the attacking and shooting areas of the pitch. This finding supports the observation by Kannekens et al. (2010) that midfielders must act as the link between a team's attackers and defenders.

We also observed an interesting retrospective activation of categories J3 (Piqué) and J15 (Ramos), two defenders with outstanding tactical and technical prowess who have an important role in the building of attacks. This finding suggests that Alonso establishes “microsocieties” in the early stages of attack by first interacting with Piqué and Ramos in a small area and then with players at the first line of attack, as shown by the prospective activation of J10 (Cesc Fàbregas) and J6 (Iniesta) in quadrant IV. These interactions reveal mastery of two skills that are of tactical significance in midfield play. The first is high proficiency in mesostructural aspects of the game, such as the ability to maintain possession of the ball in small spaces (Rampinini et al., 2007), while the second is mastery of the 360° turn (Bloomfield et al., 2007), which is a highly valued skill in a midfield player, as it enhances peripheral vision and spatial intelligence (Gardner, 2016) and allows players to adapt to the constant changes around them. To master these two skills, the player must not only excel in the technical skills required of a midfielder (Taylor et al., 2005) but also demonstrate creative and attacking qualities, such as versatility, good on-the-ball movement, and tactical positioning.

Coaches should take these factors into account in order to design effective defensive tactics. Applying pressure to Alonso and likely receivers of a pass from him when the team is in possession of the ball, for example, could help to increase the effectiveness of the defense. In addition, these pressure maneuvers could cause the observed player to move into residual spaces of the playing field away from their usual range of action, where their role within the team could move to a less important role.

### Alonso and his interaction with different areas of the pitch

As shown in the vector map in Figure 5, we detected mutual activation between Alonso and zones ZI51 and ZI71, which are two areas of creative play. Activation of these zones in quadrant I is interesting, as Alonso is largely considered to be a defensive or holding midfielder, and it further supports the idea that Alonso's role extends beyond what is traditionally expected of a defensive midfielder (Wiemeyer, 2003). The above interactions, combined with Alonso's use of space, suggest that Alonso represents a new style of midfielder, one who masters multiple aspects of offensive and defensive play.

The relationship between Alonso and areas of the pitch where attacks are launched, zones ZI10 and ZI20, was relatively unremarkable, with respective vector radius lengths of 3.18 and 2.21. These vectors were located in quadrant II, where the focal behavior activates the presence of the conditional behavior retrospectively but not prospectively. This observation is directly related to the early launch of an attack in the defensive areas of the pitch. When the backline defenders gain possession of the ball, Alonso activates his movements at the next line of play (the midfield area) in the hope of receiving the ball and moving it up to the next line of attack. Alonso's position at the tip of the triangle formed by these areas and their respective occupants does not appear to be arbitrary. Recent studies claim that these geometric patterns are consistent with Voronoi diagrams, which are diagrams based on mathematical spatial partitioning algorithms that have been recently applied to the study of soccer (Sumpter, 2016).

On analyzing the areas of the pitch where interventions involving Alonso ended, we observed that the mutual activation between Alonso and zones ZF50, ZF60, and ZF70 in quadrant I is consistent with the findings of James et al. (2002), who stated that the safety pass was one of the most frequently used technical actions by defensive midfielders. Soccer thus is not just about ball control but also about creativity and the strategic use of space, as indicated by the retrospective activation of ZF81 in quadrant IV. In view of the above findings, it is conceivable that the microsociety formed by Ramos (J15), Piqué (J3), and Alonso (J14) is designed to create space in distant areas of the pitch by drawing defenders to the passing triangle formed by these three players. The tactic is clear: the team distracts the defenders through on-the-ball interactions and then delivers the ball to the opposite side of the pitch (ZF81) to continue the attack. Essentially, this tactical play involves changes of direction, which were described in Garganta's (1997) study of offensive tactics in elite soccer some 20 years ago. Distraction and dummy moves are two important tactics employed by Alonso.

At a practical level, coaches looking to defend against such tactics should focus on studying their rivals' use of space. Placing more defenders in strategic areas, including those located some distance from the ball, will improve a team's chances of a successful defense.

### Alonso's role in game situations (interruptions and interceptions)

Versatility in set-play situations is one of Alonso's greatest attributes, as indicated by the mutual activation observed between Alonso and GKTO (goal kick for Spanish team) and GKATO (corner kick against Spanish team) in quadrant I. Alonso excels in aerial play and is a particular good header. This attribute is particularly interesting for the Spanish team, which had the shortest average squad at UEFA Euro 2012. The most interesting observation related to the association between Alonso and set-play situations was the retrospective inhibitory relationship in quadrant II, which showed that Alonso tended to be the first player to receive the ball after an interruption of play. We believe that this finding is interesting for two reasons. First, Alonso would appear to be the player of choice to receive the ball following a set play and second, it supports claims by Fransen et al. (2016) that players in central positions are the most prominent players in the building of an attack due to their ability to coordinate the flow of information between the different members of the team. In this case, Alonso would appear to be responsible for triggering the flow of information that then diffuses through the other members of the team.

At a practical level, placing more defenders on Alonso could limit his freedom to move and the time he has to take decisions. Another possible action would be a possible individual marking on the player.

### Alonso and technical actions

Based on the data from quadrant III, where focal and conditional behaviors are mutually inhibitory, we cannot draw any conclusions on the relationship between Alonso and technical actions during UEFA Euro 2012. Our findings in general, however, illustrate that Alonso draws on a wide repertoire of technical resources that are not typically associated with midfield players (Wiemeyer, 2003; Taylor et al., 2005; Thelwell et al., 2006), and is closely involved in set plays, such as free kicks and corner kicks. Alonso is a multidisciplinary and highly skilled player. The decision on which technical action to execute needs to be taken fast, but this speed of decision needs to be accompanied by accurate execution, which in turn requires precision of both motor and cognitive skills. It is this fine balance that makes a great midfielder. We agree with Kannekens et al. (2009) that soccer training models require a paradigm shift and need to center on tactical aspects of the game. Broadly speaking, we suggest the incorporation of training drills focused on teaching players to take fast, innovative decisions in pressure situations in order to adapt to the changing circumstances of the game.

Increasing players' understanding of technical and tactical concepts will help defenders adapt to the new style of midfielder represented by Xabi Alonso. Tactics such as pressure, dissuasion, and timing are likely to be more effective than the traditional tackle.

### Alonso and shots

Although Alonso scored two goals at UEFA Euro 2012, our analysis did not detect any significant results for the relationship between Alonso and type of shot.

Nonetheless, we can draw on the data compiled to characterize this relationship. When the Spanish team is in possession of the ball, Alonso establishes polyvalent relationships with players in different positions, attaining thus a wide sphere of influence. In this respect, his role differs considerably from that of a classic midfielder, although he retains his defensive function. In addition, he combines rapid decision-making with high precision, as would be expected of a midfielder with a wide repertoire of skills. Finally, Alonso is the player of choice to receive the ball during set plays.

## Conclusions and future work

We have analyzed the different relationships that Alonso establishes with his teammates on the Spanish national soccer team and studied his use of space and technical-tactical skills during the course of play. In addition, we have made some practical recommendations based on our findings that could be of interest to coaches at different levels. Our study also highlights the potential of observational methodology (in particular polar coordinate analysis) as a means of studying the spontaneous behavior of players in their natural setting. Future studies should continue to analyze the qualities of Alonso and other players to further uncover on the complex structures underlying interactions between different members of a team. The findings will undoubtedly result in a greater understanding of soccer as a whole.

## Author contributions

RM: Collected the data, reviewed the literature, and wrote the manuscript; MA: Collected and analyzed data and performed statistical analyzes.

### Conflict of interest statement

The authors declare that the research was conducted in the absence of any commercial or financial relationships that could be construed as a potential conflict of interest.
